# Propulsion in Cubomedusae: Mechanisms and Utility

**DOI:** 10.1371/journal.pone.0056393

**Published:** 2013-02-20

**Authors:** Sean P. Colin, John H. Costello, Kakani Katija, Jamie Seymour, Kristen Kiefer

**Affiliations:** 1 Marine Biology and Environmental Science, Roger Williams University, Bristol, Rhode Island, United States of America; 2 Whitman Center, Marine Biological Laboratories, Woods Hole, Massachusetts, United States of America; 3 Biology Department, Providence College, Providence, Rhode Island, United States of America; 4 Applied Ocean Physics and Engineering, Woods Hole Oceanographic Institution, Woods Hole, Massachusetts, United States of America; 5 Queensland Tropical Health Alliance, James Cook University, McGregor Road, Cairns, Australia; University of Hull, United Kingdom

## Abstract

Evolutionary constraints which limit the forces produced during bell contractions of medusae affect the overall medusan morphospace such that jet propulsion is limited to only small medusae. Cubomedusae, which often possess large prolate bells and are thought to swim via jet propulsion, appear to violate the theoretical constraints which determine the medusan morphospace. To examine propulsion by cubomedusae, we quantified size related changes in wake dynamics, bell shape, swimming and turning kinematics of two species of cubomedusae, *Chironex fleckeri* and *Chiropsella bronzie*. During growth, these cubomedusae transitioned from using jet propulsion at smaller sizes to a rowing-jetting hybrid mode of propulsion at larger sizes. Simple modifications in the flexibility and kinematics of their velarium appeared to be sufficient to alter their propulsive mode. Turning occurs during both bell contraction and expansion and is achieved by generating asymmetric vortex structures during both stages of the swimming cycle. Swimming characteristics were considered in conjunction with the unique foraging strategy used by cubomedusae.

## Introduction

The functional ecology and size of medusae have been shown to be related to and predicted by bell form ([1]and references therein). The important role of bell form derives from the bell’s role in determination of hydrodynamics and energetics during swimming [2], [3], [4], [5], [6] ultimately influencing how medusae forage and their trophic role [7]. To examine how bell shape relates to size among medusan taxa, Costello and others (2008) generated a morphospace including all known medusan species. The morphospace identified two primary morphotypes common among medusan taxa: small prolate ambush predators and large oblate feeding-current predators. Medusae within each morphotype shared common bell morphologies and functional and ecological traits [1]. Furthermore, the morphospace revealed a strong relationship between medusan size and bell shape; whereby, below a size threshold of about 10 cm diameter, bell shape varied greatly – i.e.; range from highly prolate to highly oblate – but above that dimensional threshold bell shape was greatly constrained and medusan bells were almost exclusively oblate in shape. Furthermore, they showed that this constraint was a physiological constraint that resulted from the interaction between bell shape and the force balance necessary for swimming [1], [8]. However, outliers existed that did not conform to the size constraint and seem to defy physiological limits. These outlying medusae were almost exclusively cubomedusae (or box jellyfish).

Dabiri and others (2007) developed a model to examine the physiological constraints that resulted in the observed morphospace. A premise of this force balance model is that bell shape (quantified as fineness ratio (f) where f = bell height/diameter) affects the force balances necessary for swimming because bell shape influences the propulsion mode of medusae. Medusae with prolate bells (f >1) swim via simple jet propulsion and those with oblate bells (f <1) swim using the more hydrodynamically complex rowing propulsion [3], [4]. To swim using jet propulsion, medusae expel the fluid inside their subumbrellar cavity at a high rate to produce sufficient thrust to overcome drag. The force required of the bell during contraction to produce sufficient thrust to swim increases as a cube of the bell diameter because it relates to the volume of the bell cavity. However, the actual force generated by the bell during contraction increases only as a linear function of the diameter because of the limits on muscle thickness of cnidarians [1], [8]. Consequently, at around 10 cm jet-propelled medusan bells are unable to generate the force required to overcome drag. Oblate shaped medusae which use rowing propulsion do not have the same force requirements for swimming and are not similarly constrained by size [8].

Few species possess morphologies that do not conform to the constraints model. However, there are some very large prolate cubomedusan species that are effective swimmers despite having bell diameters that surpass model predictions. These medusae swim with a pulse frequency of ∼1 Hz and appear to be jet propelled. If the physiological limits described for jet-propelled medusae are correct, then large cubomedusae must either have a unique muscle structure that increases the forces produced by the muscles or, despite their shape, swim using rowing propulsion. The muscle structure of cubomedusae has been described and shown to have the same basic epitheliomuscular structure as other medusae [9]. Since muscle structure is an unlikely explanation, we hypothesized that, even with prolate bell shapes, large cubomedusae swim using rowing propulsion and, consequently, their bell dimensions are not constrained by the cnidarian limitations of jet propulsion.

Cubomedusae have several other traits that are unique among cnidarian medusae. The most well-known unique trait is their complex, lensed eyes. While their eyes have been described previously [10], recent studies are beginning to clarify the unique capabilities of these eyes for avoiding objects [11], [12], color and spatial discrimination [13] and orienting to terrestrial visual cues [14]. In addition, cubomedusae are unique among medusae in the potency of the toxins discharged by their nematocysts and in their swimming speed and maneuverability [15]. All of these traits presumably favor effective foraging by cubomedusae in their native habitats as they prey upon large crustaceans and small fish [16], [17], [18].

The goal of this study was to quantify the influence of bell size on body kinematics and fluid interactions of cubomedusae during development. These swimming traits are evaluated relative to other medusae in light of the unique cubomedusan requirements for foraging.

## Methods

We collected *Chiropsella bronzie* and *Chironex fleckeri* in January 2009 at James Cook University in Cairns, Australia. *C. bronzie* were hand-collected from marina docks and *C. fleckeri* were collected by seining in the intertidal zone along local public beaches. No permits were required for these collections and no permission was needed to sample in these locations. The collected medusae ranged in size (interpedalial distance) from 0.5–5.6 cm (*C. bronzie*) and 2.0 to 16 cm (*C. fleckeri).* All medusae were subsequently transported to the laboratory and maintained in healthy condition in large aquaria during the experiments.

Swimming kinematics and fluid interactions during swimming were measured for individuals placed into a large aquarium containing filtered seawater seeded with hollow glass spheres (10 µm). Medusae were then illuminated using a 680-nm wavelength laser sheet and recorded at 500 frames s^−1^ using a high-speed digital video camera (Fastcam 1024PCI; Photron) placed perpendicular to the laser sheet. The laser sheet illuminated a two-dimensional plane of fluid, and data were collected when the center of the medusa bell was bisected by the laser plane. Fluid velocities were determined using digital particle image velocimetry (DPIV) software package (DaVis, Lavision Inc.) that analyzed sequential video frames using a cross-correlation algorithm. Image pairs were analyzed with shifting overlapping interrogation windows of decreasing size (64 × 64 pixels then 32 × 32 pixels). This analysis generated velocity vector fields around the swimming medusae. Velocity and vorticity field data were exported from DaVis and later used to calculate various fluid quantities using an in-house Matlab code. Bell kinematics were quantified from the cross-sectional images of the bell and were analyzed using Image-J software (NIH).

Bell and swimming kinematics were quantified using the same methods as Colin and Costello (2002). Changes in bell shape were quantified using the fineness ratio, f, defined as
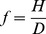
(1)the ratio of the bell height (*H*) and diameter (*D*). The swimming speed, *U*, was found by selecting the apex of the bell in each consecutive image, and dividing the bell’s displacement between frames by the inverse of the camera’s frame rate (or 0.002 s).

To evaluate the propulsive efficiency of medusae, we calculate the Froude efficiency, a common metric used to compare swimming across organisms. The Froude efficiency, *η*, is the ratio of the mechanical work resulting in forward motion to the total work expended during swimming, and can be expressed as
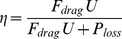
(2)where *F_drag_* is the hydrodynamic drag, *U* is the swimming speed and *P_loss_* is the power loss or the flux of kinetic energy (KE) into the wake per unit time. *P_loss_* is the excess *KE* in the wake divided by the contraction time of the medusae (*t_c_*). KE is calculated by revolving one half of the velocity field such that
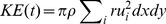
(3)where dx and dy correspond to the velocity field mesh size, r is the distance from the velocity vector to the axis of revolution, ρ is the fluid density (for seawater at 20°C, ρ = 1020 kg m−3), and ui is the velocity vector. The subscript i denotes a single velocity vector, and KE is found by summing all of the contributions of KE from each vector ui in the velocity field.

The hydrodynamic drag acting on the medusa’s body can be found from the drag coefficient, *C_d_*, by
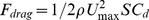
(4)where *S* is the surface area of the bell (estimated as a hemiellipsoid), *U_max_* is the medusae’s maximum swimming speed, and *C_d_* is the drag coefficient. Values for *C_d_* can be found from empirical models that are dependent upon the Reynolds number (Re* = UD/ν)* of the swimming medusa, where *D* is the bell diameter and ν is the kinematic viscosity of seawater (ν = 1.05 × 10^−6^ m^2^ s^−1^ at 20°C). Two empirical models for *C_d_* were used to evaluate the hydrodynamic drag. The first model was developed [19] and is applicable for Re >100



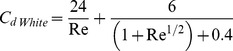
(5)The second model for drag coefficient [20] is dependent on the shape of a streamlined object operating within the range 10^3^< *Re* <10^6^

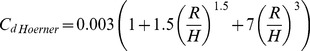
(6)where *R* and *H* are the radius and height of the medusan bell, respectively. Since the *Re* for swimming cubomedusae ranges from 10 to 10000, these empirical models can be applied to determine hydrodynamic drag with some certainty.

Analyses of wake structures were performed using DPIV velocity vector fields. The contribution of different fluid regions to the starting vortex during bell contraction was measured as the momentum flux (p) of fluid across transects positioned at different locations relative to the bell and starting vortex ring. Due to shadowing, only the vortex ring on one side of each medusa corresponding to the side of incident light was analyzed. Prior to DPIV analysis, the video frames were reoriented so that the trajectory of swimming medusae was parallel to the y-axis. With this orientation, the velocity of the jet of fluid emerging from the subumbrellar cavity had a predominantly y-component (*u_y_*). In contrast, the velocity of the fluid entrained from outside the bell past the bell margin had a predominantly x-component (*u_x_*). The momentum flux of the jet (p_jet_) was calculated using the total velocity of the fluid (*u_T_*) and its associated y-component (*u_y_*) extracted along a transect extending from the center of the starting vortex to the center of the jet at mid-contraction as:
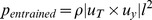
(7)where ρ is the density of seawater and *l* is the length of the interrogation square of the vector field. To estimate the momentum flux of the entrained fluid (p_entrained_) the total velocity of the fluid and its x-component were extracted along a transect that extended from the center of the vortex ring to the bell margin at the same period in the contraction cycle as that of the jet (Eq. 7) as:



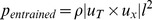
(8)To examine how the medusae manipulate fluid during turns we tracked the movement of the starting vortex rings and the total circulation of the rings (Γ). Circulation was quantified as:
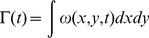
(9)where ω is a single value of vorticity within the vorticity field.

## Results

**Figure 1:**
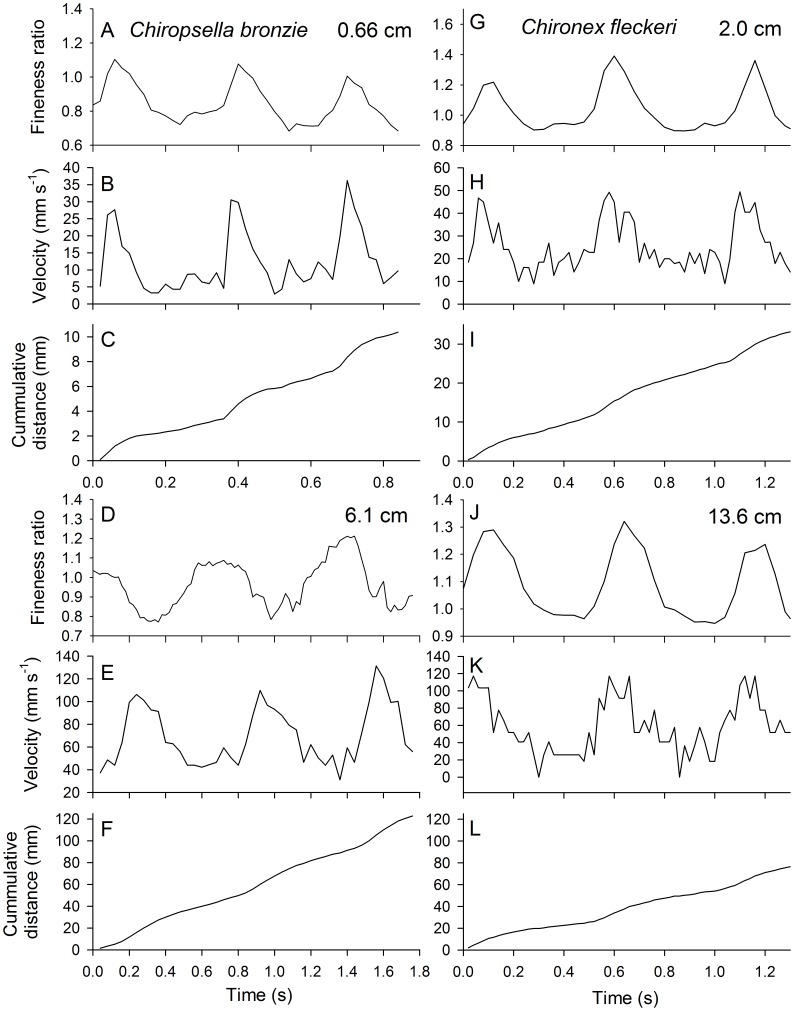
Swimming kinematics of small (a–c and g–i) and large (d–f and j–l) *Chiropsella bronzie* and *Chironex fleckeri*. Fineness ratio illustrates changes in bell shape throughout the swim cycle where peak fineness corresponds with maximum bell contraction. Peak velocities are achieved during bell contraction but there is a small increase in velocity also observed at end of bell expansion. Regardless of size, the medusae maintain continuous forward progress throughout the pulse cycle.

**Figure 2:**
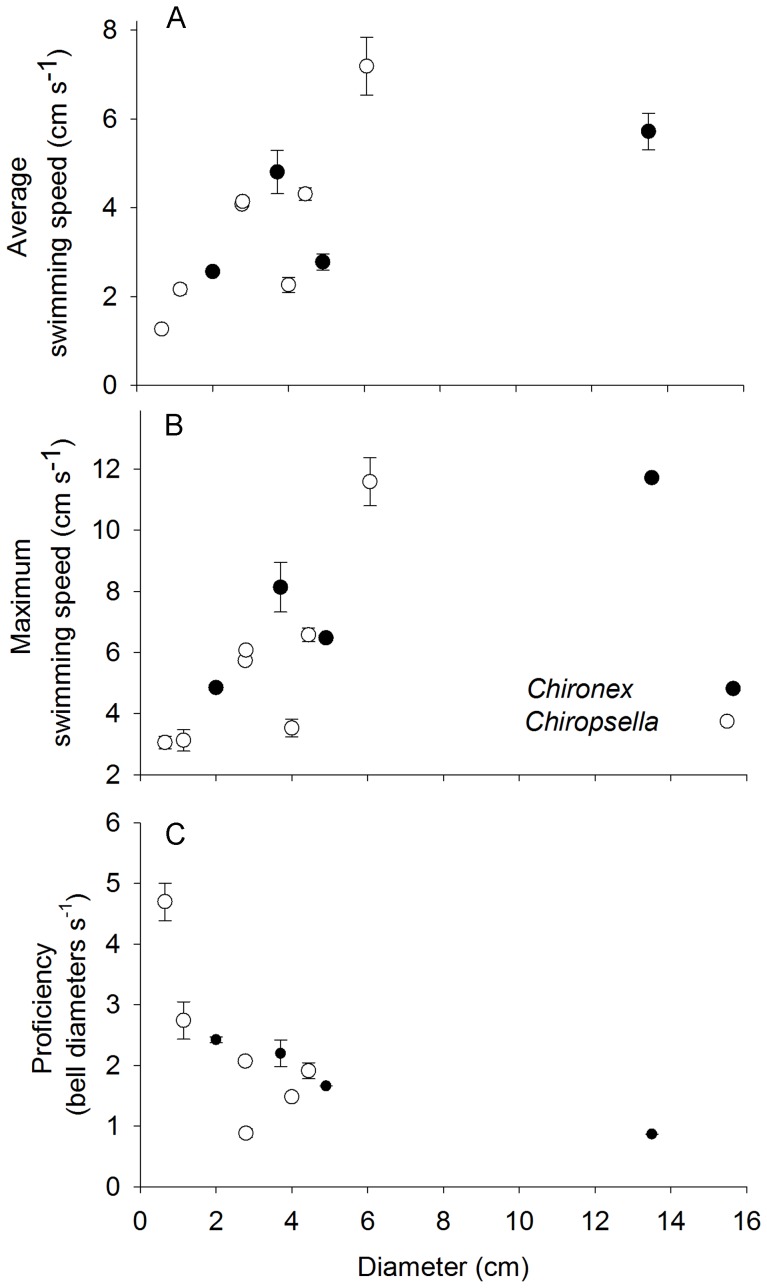
Change in swimming performance with size. Data are mean (± st. dev.) of three consecutive swimming cycles.

Swimming kinematic profiles illustrate how the bells contracted (increasing fineness ratio) and expanded (decreasing fineness ratio) during consecutive swim cycles and the forward progress of the medusae over time (Fig. 1). They reveal that both species pulsed rapidly at 2–3 Hz depending on their size with only brief pauses between consecutive pulses (Fig. 1). As with most medusan species, the smaller medusae pulsed more rapidly than the larger medusae (Fig. 1). But unlike many medusae, even the smallest cubomedusae made continuous forward progress, and always maintained positive swimming velocities, even during bell expansion. With each pulse the medusae achieved high peak velocities. Unlike rowing hydromedusae [21], the average and peak velocities of both cubomedusan species increased with their size (Fig. 2a and b). However, their proficiency (swimming velocity normalized by bell diameter as defined by Dabiri et al 2010) decreased as the cubomedusae increased in size (Fig. 2c).

**Figure 3:**
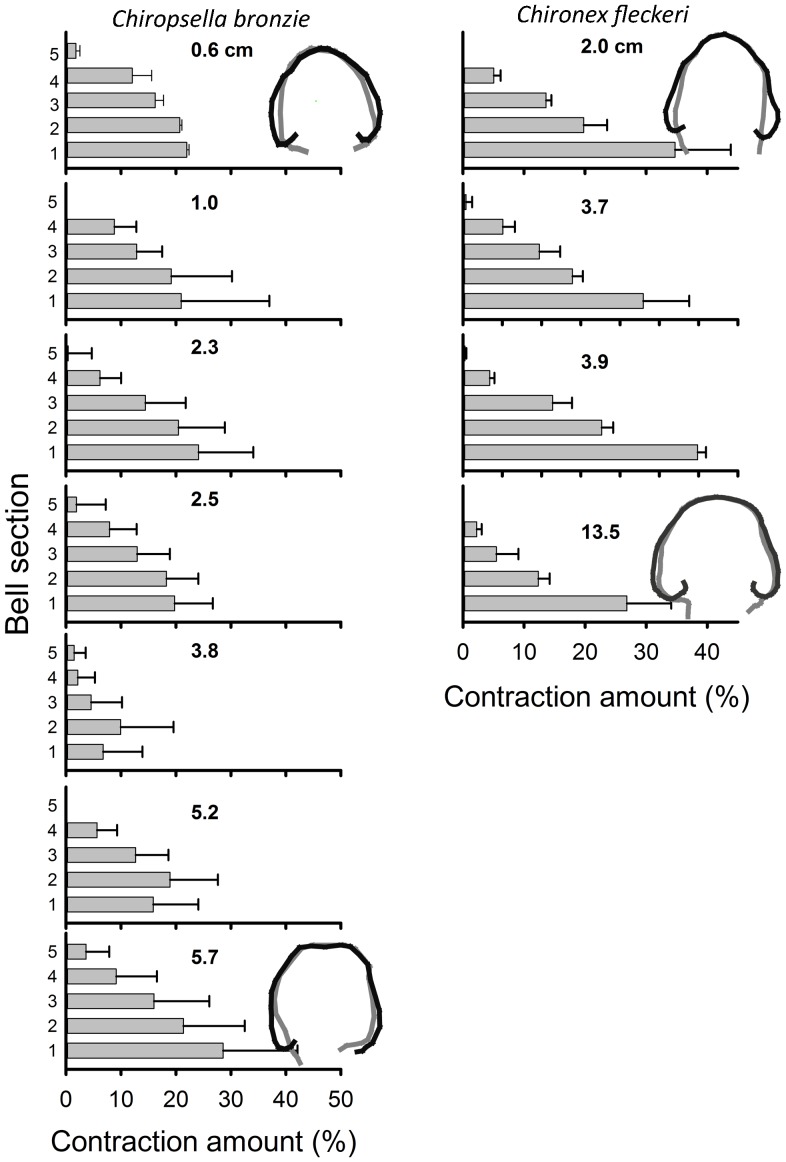
Percent change in diameter of bell sections for different sized *Chiropsella bronzie* and *Chironex fleckeri*. Lengths indicated in cm. for each figure represent maximum bell diameters during relaxation. Values represent mean (± st. dev.) diameters among three consecutive swimming cycles. Sketches are bell outlines during maximum expansion (black) and contraction (grey) for the smallest and largest individual examined of each species.

To examine how bell kinematics change through development we quantified how much different sections of the bell contracted relative to the position at rest (Fig. 3; [22]). For cubomedusae of all sizes, the magnitude of the bell contraction was greatest at sections close to the bell margin. In fact, there did not appear to be any developmental alterations in the location or magnitude of bell contraction. However, the overall magnitude of the bell contraction did vary among some individuals and may have reflected different swimming strides.

**Figure 4:**
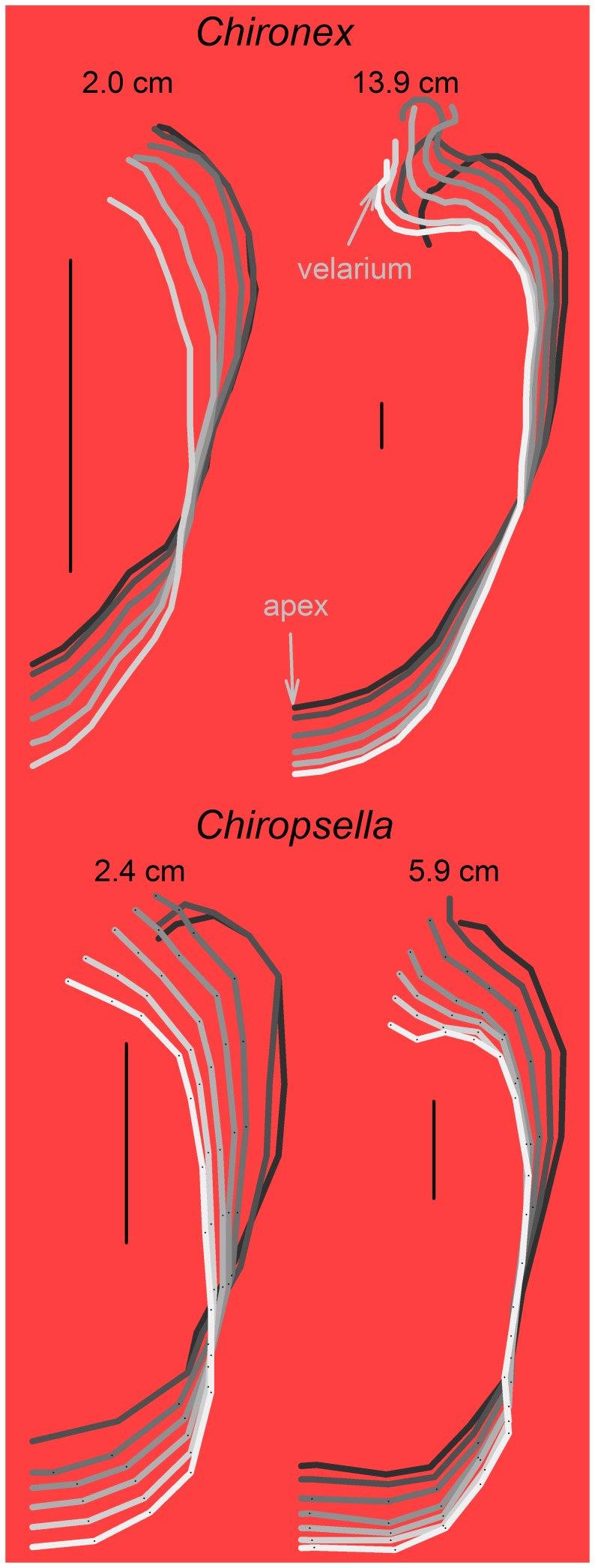
Outlines of the bell and velarium at equal time intervals during bell contraction for small and large *Chironex fleckeri* and *Chiropsella bronzie*. Black outline is the initial bell shape and white is the final shape at maximum bell contraction. Only half of the bell is shown with the apex on the bottom and bell margin along the top. Velarium of the smaller medusae have simple outpocketing kinematics while the velarium of larger medusae have more complex kinematics and greater inflexions.

**Figure 5:**
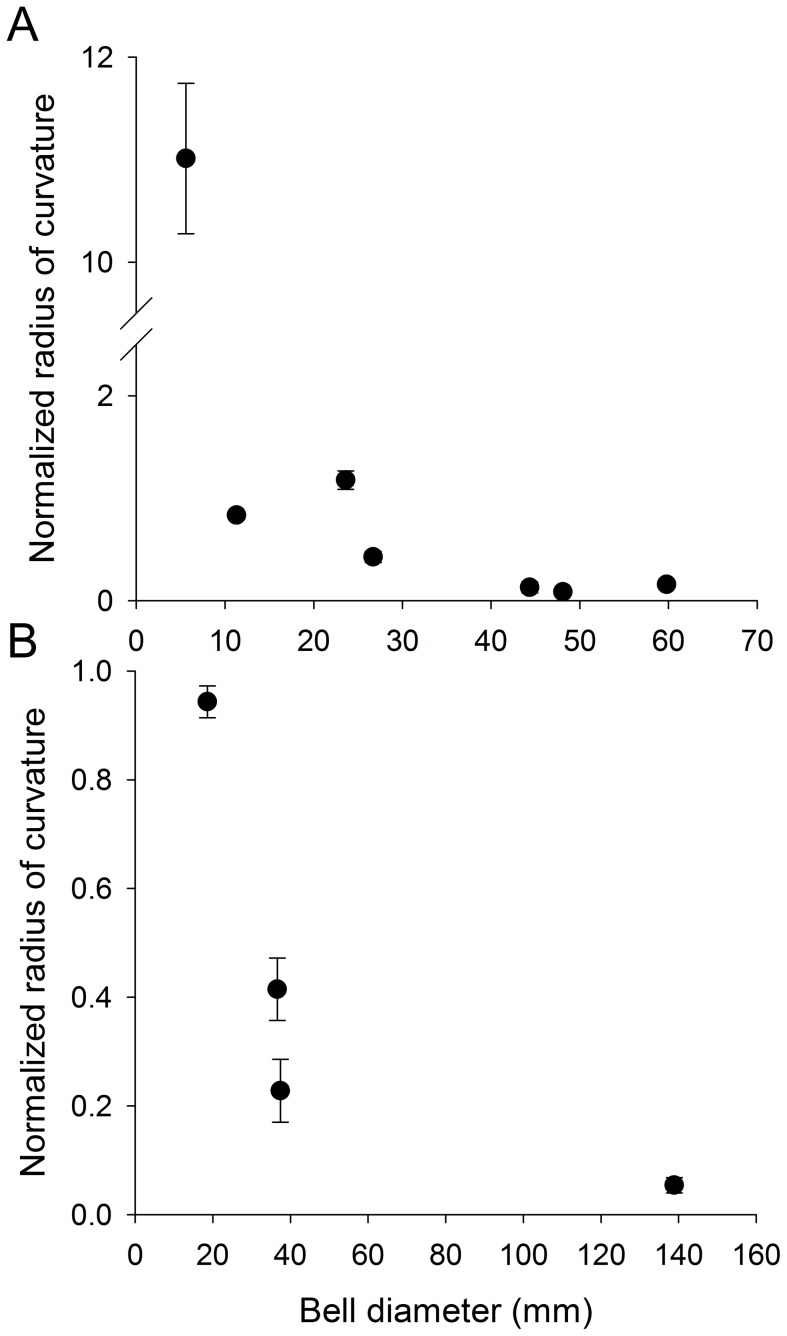
Normalized radius of curvature (normalized by bell diameter) of the velarium at maximum bell contraction of *Chiropsella bronzie* (A) and *Chironex fleckeri* (B). Radius of curvature is inversely related to inflexion of a surface; flattened or low inflexion surfaces possess high radii of curvature whereas highly curved or flexed surfaces are characterized by low radii of curvature. Small cubomedusae exhibit little bending of the velarium during bell contraction and consequently have high normalized radii of curvature. In contrast, as cubomedusae increase in bell diameter, the velarium bends more extensively during bell contraction and results in lower normalized radii of curvature.

While there were not any conspicuous alterations in bell contraction through development, the kinematics of the velarium did differ between small and large cubomedusae (Fig. 4). For smaller cubomedusae, the velarium was simply pushed out of the subumbrellar cavity by the expelled fluid during contraction and then sucked back into the cavity by the fluid during expansion. For large individuals, the kinematics of the velarium were more complex. During the initial stages of the contraction the velarium was pushed out of the subumbrellar cavity. However, subsequent velarium kinematics were characterized by a traveling wave of inflexion as the velarium swept inwards toward the bell midline (Fig. 4). Velarium inflexion, or bending, traveled from the bell margin to the tip of the velarium during bell contraction. This extensive flapping motion in large individuals is evident in the greatly reduced radii of curvature of the inflexion of the velarium for different sized medusae (Fig. 5). In contrast, the velaria of small medusae during bell contraction were quite straight with relatively large radii of curvature.

**Figure 6:**
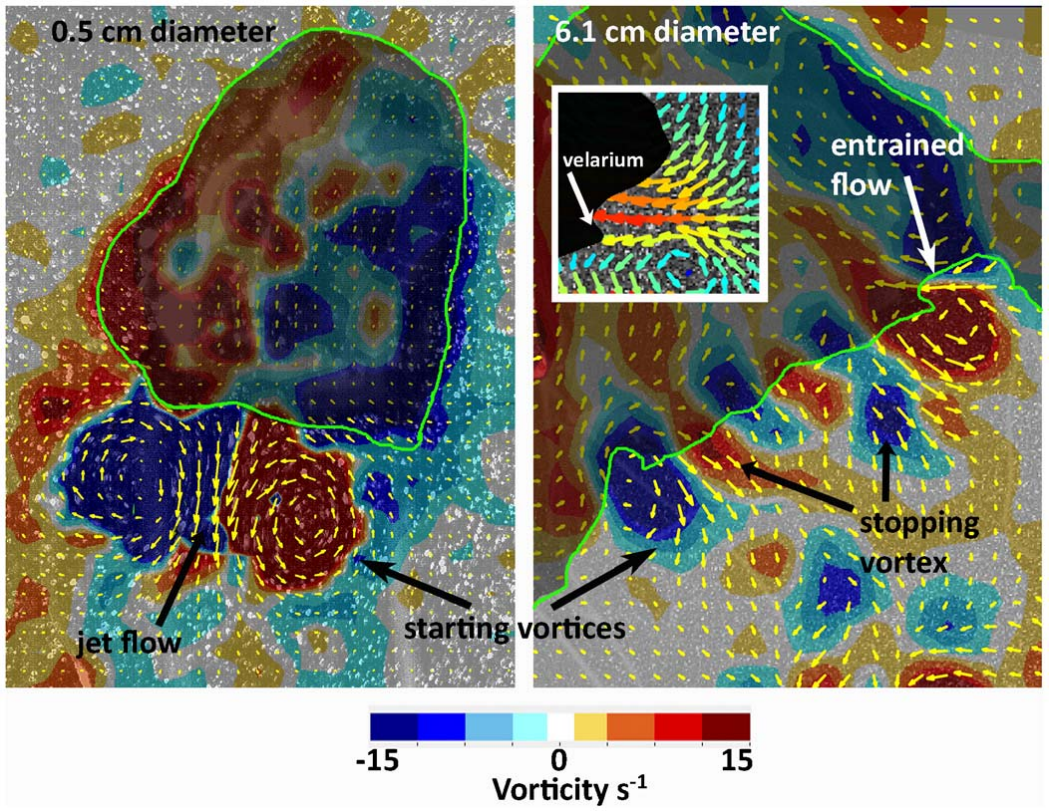
Vorticity contour and velocity vector plot of flow during maximum contraction for a small and large *Chiropsella bronzie*. Flow emerging from the bell of the small medusa is characterized a simple jet (i.e.; jet of fluid and a single vortex ring). Flow generated by the large medusa is more complex with the stopping vortex from the previous swim cycle interacting with the starting vortex generated during bell contraction and flow entrained from the bell margin by the velarium (inset) contributing to the starting vortex. Inset: close-up of entrained flow in region adjacent to velarium. Note that the maximum velocities (red) are oriented toward the inflexion point of the velarium.

**Figure 7:**
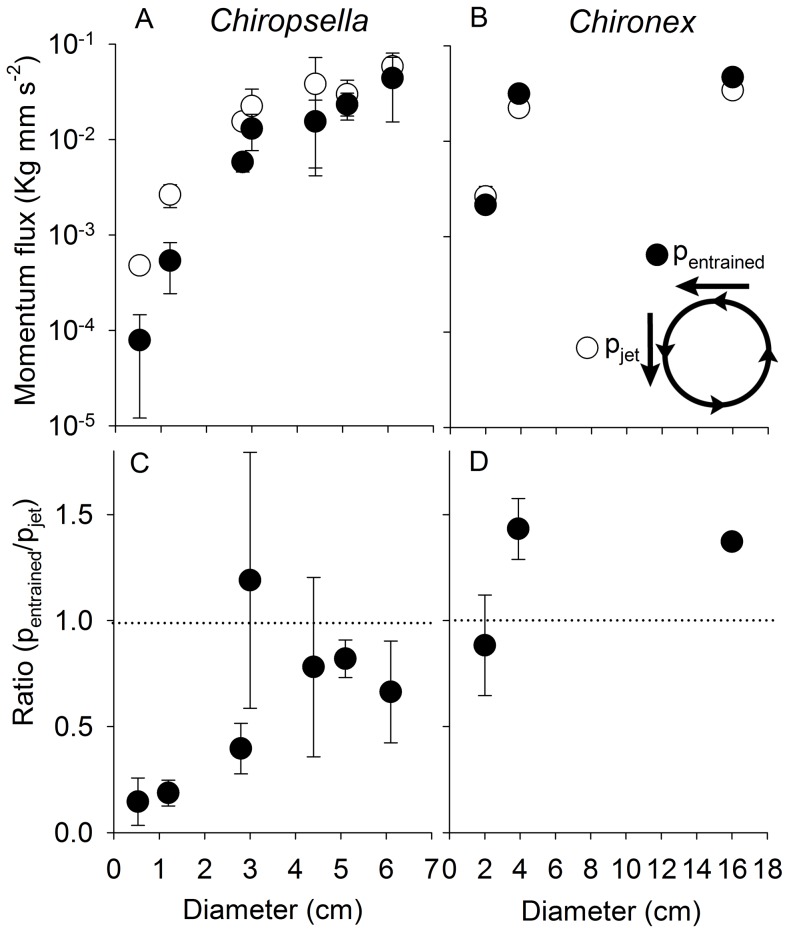
Momentum flux (A and B) and relative momentum flux (C and D) of fluid entrained at the velarium and the jet flow from the bell. As the size of *Chiropsella* increased the contribution of the entrained flow to the starting vortex increased. The contribution of the entrained flow for *Chironex* was large at all sizes but also increased with bell diameter.

Developmental differences in velarium kinematics were accompanied by changes in medusan wake dynamics. The most conspicuous features within wakes of small medusae were strong jet flows expelled from the subumbrellar cavity during contraction (Fig. 6). A similar jet was also observed for larger medusae but, in addition, high fluid velocities were characteristically present outside the bell along the bell margin. The peak velocities of the flows from outside the bell margins of larger medusae were associated with, and directed toward, the inflexion points of the velarium (Fig. 6 inset). A comparison of the momentum flux of the jet emerging from inside the bell with that of the entrained fluid from outside the bell demonstrated that the contribution of the entrained fluid increased in the wake with medusan size (Fig. 7). For both species, the momentum of the entrained fluid was nearly equal to that of the jet fluid by 4 cm diameter. This suggests that the momentum contributing to thrust during bell contraction changed during development of the cubomedusae. For smaller medusae (<4 cm) thrust almost exclusively came from the jet emerging from inside the bell. In contrast, thrust for larger medusae came from a combination of the jet expelled from inside the bell and fluid entrained from outside the bell. The combination of these two mechanisms is characteristics of rowing propulsion [3], [4].

The wake structures of the different propulsive modes used by small and large medusae also highlight key distinctions between jetting and rowing propulsion (Fig. 6). Vorticity fields revealed differences in the sense and magnitudes of fluid rotations within wake vortices. For small medusa, only the starting vortex ring was visible in the wake and the maximum jet velocities were located within a single region - the interface of opposite-rotation regions within the starting vortex ring. However, the wakes of the larger rowing medusa consisted of adjoining starting and stopping vortex rings expelled simultaneously during bell contraction. For these medusae, maximum wake velocities also occurred at opposite-rotation interfaces, but these opposite-rotation interfaces were located at positions of starting-stopping vortex interfaces. Consequently, cross-sections of larger medusan wakes contained two regions with high velocities because starting-stopping vortex interfaces occurred on either side of the center of the wake (Fig. 6).

**Figure 8:**
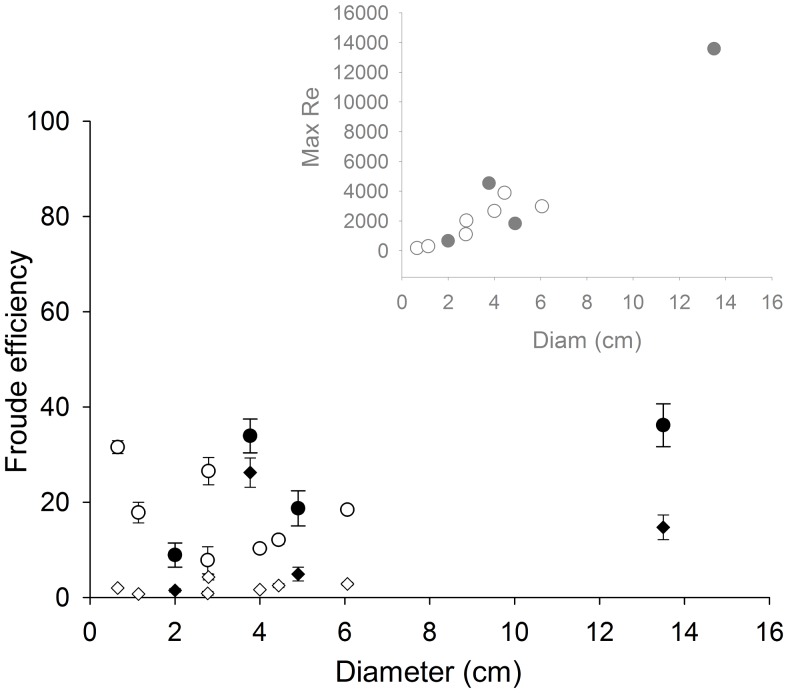
Effect of medusan size on the Froude efficiency of *Chiropsella bronzie* (open symbols) and *Chironex fleckeri* (closed symbols). Two different models of drag were used: White (circles) and Hoerner (diamonds). Inset: Changes in Reynolds number of flow around the bell with medusan diameter.

While effective for generating high swimming speeds, the propulsive strategy of the *C. bronzie* and *C. fleckeri* result in Froude efficiencies no greater than 40% and many estimates are below 20% (Fig. 8).

**Figure 9:**
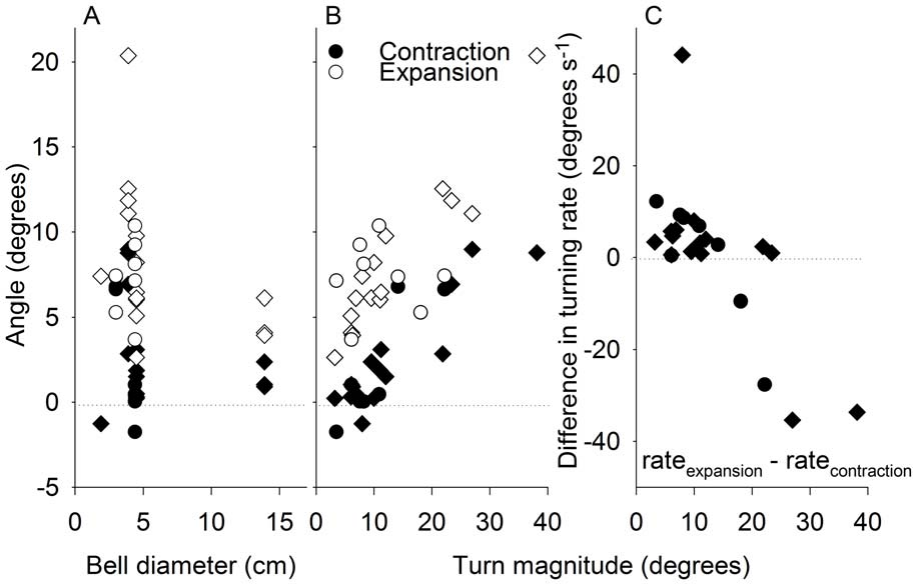
Comparision of turning dynamics during bell contraction and expansion of *Chiropsella bronzie* (circles) and *Chironex fleckeri* (diamonds). (A) The effect of medusan size on turning angle during bell contraction and expansion. (B) The effect of total turn magnitude on turning angle during bell contraction and expansion. (C) The effect of total turn magnitude on turning rate during contraction and expansion. The difference in turning rate (rate_expansion_ – rate_contraction_) was calculated to illustrate the relative difference between the two phases. Positive versus negative values indicate the bell mostly turned during expansion versus contraction, respectively.

In addition to swimming at high speeds, cubomedusae are also highly maneuverable. Both species turned during both bell contraction and bell expansion (Fig. 9a and b) and the swimming cycle phase used for turning did not differ with animal size (Fig. 9a). However, for smaller turns the medusae primarily turned during the expansion phase (i.e.; recovery stroke) of the swimming cycle. As the magnitude of the turn increased, however, the degree of turning increased during the contraction phase (i.e.; power stroke). Consequently, sharp turns involved turning throughout the swimming cycle (Fig. 9b). However, since the contraction time is generally much shorter than the expansion time (Fig. 1), the turning angle may be a misleading measure of swimming cycle differences in turning. Therefore, we also calculated the turning rate (turning angle/contraction or expansion time) and found that phase dependence of turning varied with the extent of the turn. Based on this measure, medusae turned faster during the expansion phase of small turns but faster during the contraction phase of for larger turns.

**Figure 10:**
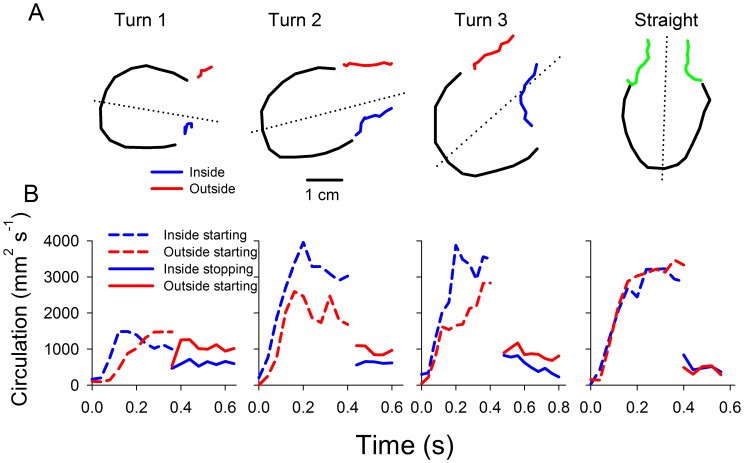
Characterization of wake vortices during turning versus straight swimming. The starting vortex is formed during bell contraction and the stopping is formed during bell expansion. (A) Path traveled by the vortex ring core on the inside (blue) and the outside (red) of turn during turning and straight swimming. (B) Circulation of the starting and stopping vortex rings on the inside and outside of the turn.

Vortex formation patterns reflected bell kinematics during turning. For straight swimming, neither the magnitude (i.e.; circulation) nor the trajectory of the starting and stopping vortices differed on either side of the bell (Fig. 10). However, during turns, the circulation magnitude and centroid trajectory of starting vortices differed on the inside versus outside of the turn. Likewise, the circulation magnitude of the stopping vortices differed on either side of the bell during turning. Specifically, the starting vortex at the bell margin on the inside of the turn during bell contraction was directed toward the central axis of the bell. In contrast, the starting vortex on the bell margin located on the outside of the turn was directed straight downstream. For the three turns examined, the circulation of the starting vortex was greater on the inside of the turn and the circulation of the stopping vortex was greater on the outside of the turn. Consequently, it appears that medusae modulate both the magnitude and the trajectory of their vortex rings during turning maneuvers.

## Discussion

The morphology, sensory capabilities, behavior and propulsion of cubomedusae are unique among medusae. However, their distinctive swimming traits appear well suited to their foraging strategies. Cubomedusae orient toward environmental cues [11], [12], [14], [23], [24] that enable them to forage in habitats with high prey densities [17], [25], [26], [27]. While medusae from other cnidarian classes are capable of sensing and responding to environmental cues (e.g. [28], [29] ), their overall energy acquisition relies on bulk processing of fluids and random encounters with prey. Consequently, their capture surfaces are designed to capture the most abundant prey or prey with the highest probability of encounter. These differences underscore the differences that are observed between cubomedusae and other medusan taxa.

For example, rowing scyphozoans, such as *Aurelia* spp. and *Cyanea* spp., and hydrozoans, such as *Aequorea victoria*, feed as feeding-current foragers [30] by continuously pulsing their bells and entraining large volumes of fluid past their capture surfaces [2], [7], [31]. As a result, their bell morphologies and propulsive modes are designed such that they swim efficiently compared to medusae with other foraging modes [6], [32] and entrain large volumes of fluid throughout their swimming cycle [2], [4], [31]. This mode of foraging is effective at encountering a wide variety of slower swimming prey [7] and some faster swimming prey [33], [34], [35]. Consequently, their capture surfaces contain a broad range of nematocyst types that are responsive to a broad range of cues and, therefore, are relatively unselective [36], [37]. While feeding-current foraging medusae may have some capacity to orient in higher prey density regions, such as frontal boundaries [28], they rely on processing large volumes of fluid to acquire sufficient energy [30] and, consequently, populations of feeding-current foraging species may be capable of processing the entire water column on the order of hours or days [38], [39].

Ambush predatory hydromedusae can similarly be considered random foragers. Prey encounter by ambushing medusae such as *Sarsia tubulosa*, *Aglantha digitale* and *Leuckartiara* sp. involves motionless fishing with extended tentacles while waiting for prey to swim into their tentacles. Consequently, ambush foraging medusae typically feed only when they not swimming [40]. For these medusae, jet propulsion is well suited to reduce the time between feeding bouts by enabling rapid repositioning in the water column. However, their speed comes at an energetic cost [6]. In contrast to feeding current foragers, the nematocysts of ambushing medusae are more selective [36], [41] and respond primarily to the prey types the medusae are most likely to encounter during ambush foraging – i.e.; large and rapidly swimming zooplankton.

**Figure 11:**
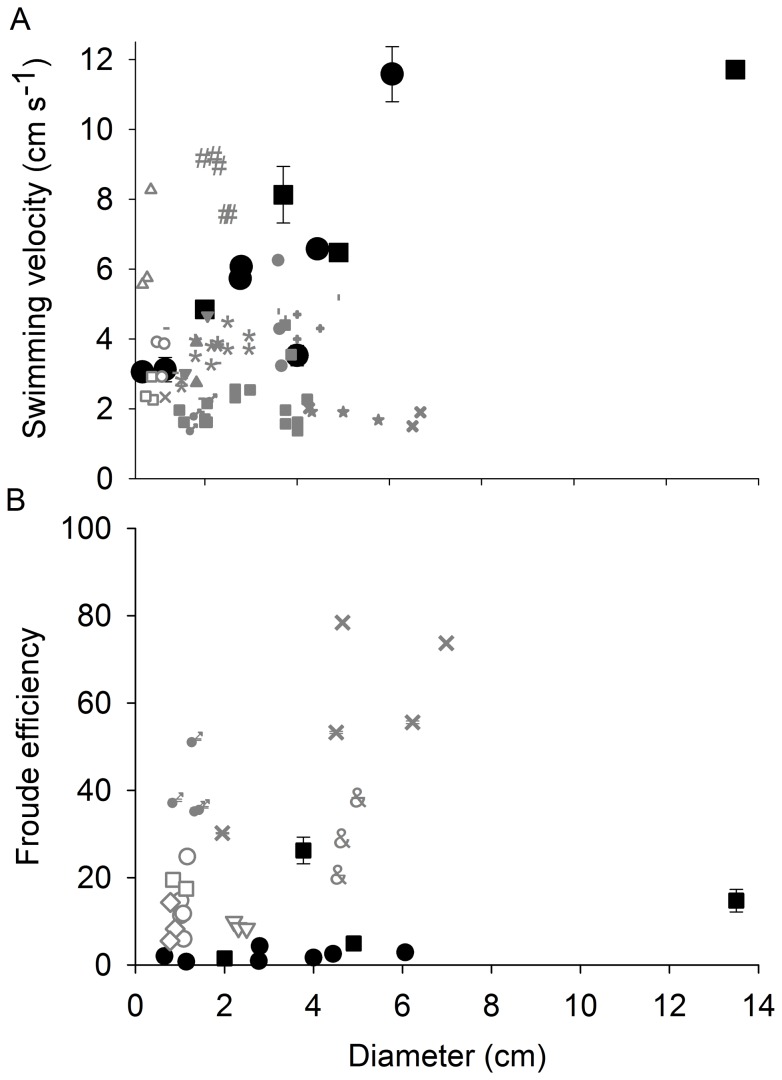
Comparison of swimming speed (A) and Froude efficiency (B) among jetting (open symbols), rowing (grey symbols) and cubomedusae (black symbols). For velocity data jetting species include: *Aglantha digitale* (Δ), *Leuckartiara* sp.(#), *Neoturris* sp.(○), *Sarsia tubulosa* (□); rowing species include: *Cassiopea* sp.(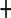), *Cotylorhiza sp* (▴), *Sandaria* sp. (▾), *Chrysaora quincirrha* (•), *Aurelia aurita* (▪), *Phyllorhiza punctata* (*), *Mastigias papua* (–), *Aequorea victoria* (&), *Mitrocoma cellularia*(x), *Phialidium gregarium* (♂), Solmissus (I), *Craspedacusta sowerbyi* (X); cubomedusae: *Chiropsella bronzie* (•) and *Chironex fleckeri* (▪).

In contrast to these other taxa, the sensory capabilities of cubomedusae – i.e. image forming eyes – have been shown to enable them to orient to objects in and out of the water [12], [42], [43]. This enables them to forage as active cruising predators (as defined by [30]) and gives them the ability to orient to and forage in specific habitats with high prey densities making cubomedusae not bulk processors of prey but rather hunters of prey. As a result of being capable of remaining in areas of high prey concentrations, their encounter rates with prey would be greatly enhanced [44]. Orienting toward and foraging in specific prey habitats, including mangroves and the littoral zone, requires a high level of swimming control and performance. A comparison of cubomedusan swimming velocities with other medusan taxa demonstrates that large cubomedusae swim more rapidly than any other medusae. Small cubomedusae do not perform as well as some jetting hydromedusae and it appears that only at large bell diameters are cubomedusae capable of the highest observed velocities among the medusozoa (Fig. 11a). To achieve these high velocities, large cubomedusae utilize a hybrid mode of propulsion. A strong jet is always produced during bell contraction (Fig. 6) but, as Gladfelter (1973) noted, that jet only comprises a fraction of the wake. If solely dependent upon this jet, large cubomedusae would be unable to produce sufficient thrust to overcome the drag forces which resist acceleration [1], [3], [8]. To augment this jet, large cubomedusae have highly flexible velariums (Fig. 4) which, during bell contraction, entrain large volumes of fluid from outside the bell (Fig. 6 inset), contributing to the momentum, and therefore thrust (Fig. 7), imparted to the fluid during bell contraction. In essence, it appears that the velarium is functioning like the flexible bell margins of rowing scyphomedusae. The flexibility of the velarium serves to accelerate fluid along the bell margin and enhance total thrust production (Colin et al, In press).

A comparison of the swimming efficiency of cubomedusae to other medusan taxa suggests that cubomedusan swimming, while effective, is not efficient (Fig. 11). Other medusae such as ambushing hydromedusae also have high energetic costs associated with swimming but they only swim a minor fraction of the time (<5%). In contrast, cubomedusae are known to swim continuously while foraging [10], [45]. As a result, cubomedusae have higher respiratory costs than other medusae [17], [46]. Again, it is likely their unique foraging strategy enables them to forage in specific prey habitats and to target prey with high energy content (relative to the prey of other medusan taxa) such as small fish and shrimp [16], [17], [18]. The high potency of the cubomedusan nematocysts [47], [48] also contributes to enabling them to capture large prey with high energy content. They also appear to compensate for their high propulsive costs by sleeping at night while not foraging in order to conserve energy [49].

Cubomedusan swimming characteristically entails a high level of maneuverability. Surprisingly, for smaller turns cubomedusae turned primarily during the recovery stroke and only for larger turns did they also turn during the power stroke (Fig. 9). Previously, a cubomedusa or jetting hydromedusa has been described to turn through the use of its velarium or velum, respectively, as a nozzle to direct the jet expelled during the power stroke [50], [51]. If a nozzle mechanism directed wake flows, cubomedusae should turn during the power stroke and direct their jet toward the inside of the turn. However, we demonstrate that cubomedusae primarily turn during the recovery stage and that they do not produce a jet directed toward the inside of the turn. Instead, the different sides of the starting vortex ring moved asymmetrically and the starting vortex ring on the outside of the turn traveled directly downstream (Fig. 10, red lines) while the inside vortex traveled toward the central axis of the medusae (Fig. 10, blue lines). Consequently, it appears that these vortex dynamics are more complex than a simple jet that has been redirected using a nozzle. Detailed examination of velarium kinematics of the cubomedusa, *Tripedalia cystophora*, revealed complex velarium kinematics during which contraction of the velarium on the outside of the turn was initially delayed relative to the inside margin, but subsequently accelerated much more rapidly during the latter part of bell contraction [42]. Delayed velarium kinematics on the outside of the turn are consistent with the delayed vortex ring formation we observed on the outside of the turn (Fig. 10).

Interestingly, we observed that the largest differences in the relative magnitude of the vortex circulation between inside and outside margins of a turn occurred within stopping vortices during the recovery stroke. This is important because stopping vortices have been shown to contribute forward thrust [32] and accelerate medusae during swimming [52]. We observed greater stopping vortex circulation on the outside of the turn suggesting that, during the recovery stroke, the bell is receiving greater thrust on the outside than the inside of the turn. This observation is consistent with greater turning by cubomedusae during bell expansion than contraction during most maneuvers. We suggest that, rather than a nozzle jet, cubomedusan turning involves asymmetric velarium kinematics that manipulate the magnitude and trajectory of starting and stopping vortices and result in asymmetric thrust on the inside versus outside of the turn. However, the limited number of turns that we were able to analyze and our inability to measure velarium kinematics for every medusa limited our ability to fully examine turning, particularly during the power stroke. A more focused study that is capable of connecting bell kinematics with wake structures is required to confirm the mechanics of cubomedusaen turning during the power stroke.

In conclusion, it has been shown for hydromedusae and scyphomedusae that medusan form and propulsion are tightly coupled with foraging strategy [1]. We suggest that the hybrid propulsive strategy of cubomedusae is also well suited for its unique cruising foraging strategy, whereby, it enables cubomedusae to swimming highly effectively, albeit at a cost, and to be highly maneuverable.
